# Benralizumab for anti‐tumor necrosis factor‐associated eosinophilic gastrointestinal disease in a child with ileal Crohn's: A case report

**DOI:** 10.1002/jpr3.70164

**Published:** 2026-03-12

**Authors:** Jonathan Dudzik, Alberto Pinzon‐Charry, Peter Lewindon, Fariha Balouch

**Affiliations:** ^1^ Department of Gastroenterology, Hepatology and Liver Transplant Queensland Children's Hospital South Brisbane Queensland Australia; ^2^ Queensland Paediatric Immunology & Allergy Service Queensland Children's Hospital South Brisbane Queensland Australia; ^3^ Griffith University Nathan Campus Nathan Queensland Australia; ^4^ University of Queensland, St Lucia Campus St Lucia Queensland Australia

**Keywords:** adalimumab, inflammatory bowel disease, infliximab, vedolizumab

## Abstract

Crohn's disease (CD) and eosinophilic gastrointestinal diseases (EGIDs) are distinct inflammatory entities, but eosinophilic disease may emerge as a paradoxical immune complication of anti–tumor necrosis factor therapy. We report a 12‐year‐old boy with terminal ileal CD who developed severe eosinophilic gastritis and ileitis, peripheral eosinophilia, and psoriasiform dermatitis during prolonged anti‐TNF treatment, despite CD remission. Corticosteroids, dietary modification, and sequential biologic therapy resulted in incomplete or transient responses. Following anti‐TNF withdrawal, eosinophilic disease persisted, prompting compassionate off‐label treatment with benralizumab, an interleukin‐5 (IL‐5) receptor α‐antagonist, alongside vedolizumab for CD maintenance. Benralizumab led to rapid normalization of peripheral eosinophil counts, resolution of gastrointestinal symptoms, improved food tolerance, and histologic remission at 6 months. This case illustrates persistent anti‐TNF–associated immune deviation and supports targeted IL‐5 receptor blockade as an effective strategy for refractory eosinophilic gastrointestinal disease while maintaining CD remission.

## 1 INTRODUCTION

Crohn's disease (CD) is a chronic relapsing inflammatory disorder characterized by transmural involvement of the gastrointestinal tract.^1^ Eosinophilic gastrointestinal diseases (EGIDs) are defined by pathological eosinophilic infiltration of the gut.^2^ Although traditionally regarded as distinct entities, eosinophilic gastrointestinal inflammation has been described as a paradoxical immune complication during biologic therapy, particularly tumor necrosis factor (TNF) inhibition.^3^, ^4^ This framework highlights a continuum of gut inflammation where lymphocyte‐ and eosinophil‐driven mechanisms converge, complicating diagnosis and informing emerging therapeutic strategies. Herein, we describe a child with CD who developed a complex disease course, characterized by anti‐TNF–associated immune complications.

## 2 CASE REPORT

A 12 year old boy was referred with abdominal pain, mouth ulcers, weight loss, bloody diarrhea, raised C‐reactive protein (CRP 50 mg/L; normal <5 mg/L), and raised fecal calprotectin (FC 950 µg/g; normal <50 µg/g) on a background of allergic rhinitis. Magnetic resonance enterography (MRE) revealed 6 cm of thickening in the distal terminal ileum (TI) with normal colonic architecture. His endoscopy showed Grade A esophagitis and Crohn's terminal ileitis (SES‐CD 9). Histology confirmed active chronic ileitis with no tissue eosinophilia. His CD partially responded to 8 weeks of exclusive enteral nutrition and 6‐mercaptopurine. Interval intestinal ultrasound (IUS) assessment demonstrated active disease and likely inflammatory TI stricture requiring escalation to anti‐TNF therapy. Clinical and biochemical (CRP, FC) remission was achieved after 6 months of 4‐weekly 10 mg/kg intravenous infliximab. At this time, CD activity was quiescent; however, subsequent endoscopic reassessment following prolonged anti‐TNF exposure demonstrated new prominent mucosal eosinophilia. Anti‐TNF trough levels were maintained >12 mg/L (local laboratory maximum range). The TI stricture improved on follow‐up IUS.

**Figure 1:**
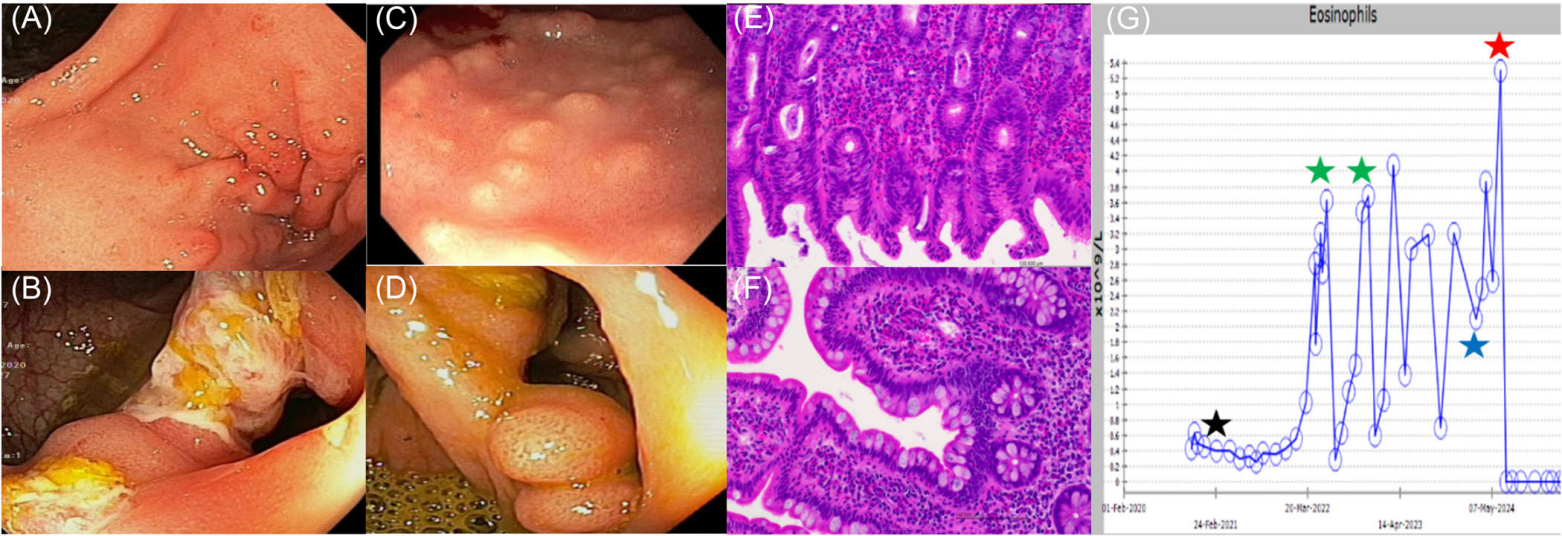
Endoscopic features illustrating evolution from CD to EGID. Initial endoscopy demonstrated (A) chronic gastritis and (B) deep ulceration at the ICV, consistent with CD. In contrast, repeat assessment showed (C) diffuse nodular gastric antral mucosa and (D) prominent ICV pseudopolyps, characteristic of EGID. Histology (H&E; x200) confirmed marked eosinophilia in (E) gastric mucosa (1192 eosinophils/mm²), and (F) terminal ileum (441 eosinophils/mm²). (G) Peripheral eosinophilia after Infliximab (black star), reduction with courses of corticosteroids (green stars), no improvement with infliximab cessation and vedolizumab initiation (blue star), and remission with benralizumab (red star). CD, Crohn's disease; EGID, eosinophilic gastrointestinal disease; ICV, ileocaecal valve.

Twelve months later, he developed mild psoriasis, nausea, and weight loss, prompting reassessment. Endoscopy showed severe nodular gastritis and ileitis. Histopathology confirmed eosinophilic gastritis (EoG) and eosinophilic ileitis (EoI) with eosinophilic infiltrate in all other upper biopsies (Figure 1). Peripheral eosinophilia of 3.63 × 10^9^/L (normal <0.5 × 10^9^/L) was demonstrated. The recommendation for a dairy‐free diet and 1 mg/kg oral prednisolone improved his symptoms. Upon steroid cessation, he developed eosinophilic psoriasis, pyoderma, and keratoconjunctivitis. Gastroscopy confirmed persistent nodular gastritis with histological mucosal eosinophilia, and skin biopsy confirmed psoriasiform lichenoid dermatitis with eosinophilic exudates.

A food elimination diet was trialled for severe vomiting triggered by previously tolerated foods, including milk, egg yolk, shellfish, chicken, and tuna, with partial response. Symptoms improved with oral prednisolone but recurred on cessation. Extensive infective, immunologic (STAT6), and genetic (Invitae primary immune deficiency panel) screens were unrevealing aside from PIBD‐associated NOD2 homozygosity, raising suspicion of an infliximab‐induced eosinophilic disorder.

An in‐class switch to adalimumab (ADA) was undertaken as a standard clinical approach to paradoxical anti‐TNF reactions, resulting in improved, but not resolved, psoriasis. However, IUS demonstrated active TI disease with ADA levels of 6.5 µ/mL (target >5 µg/mL), prompting escalation to weekly 40 mg dosing, which achieved higher trough levels and biochemical remission. Despite this, quality of life was poor due to ongoing psoriasis and vomiting, leading to an out‐of‐class switch. Vedolizumab 300 mg intravenously every 4 weeks (Figure 1; blue star) was commenced in accordance with local escalation practice and maintained CD remission, assessed by clinical symptoms, CRP, FC, and IUS. Vedolizumab was used solely for maintenance, with upper endoscopy confirming persistent gastric mucosal eosinophilia of up to 50 eosinophils/high power field.

**Figure 2:**
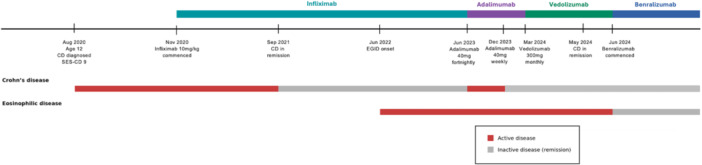
Timeline of disease course, biologic therapies, and inflammatory phenotype activity.

Benralizumab, an interleukin‐5 receptor (IL‐5) alpha antagonist, was compassionately applied for off‐label use and initiated to reduce mucosal eosinophilic burden via cell‐mediated cytotoxicity.^5^ Before initiation, the child had severe peripheral eosinophilia (5.3 × 10^9^/L) and significant esophageal and gastric EGID. His peripheral eosinophil count was undetectable after one 30 mg subcutaneous dose (Figure 1G; red star). Marked improvement in food tolerance and vomiting was noted after his third dose whereby his frequency interval was de‐escalated from 4‐weekly to 8‐weekly. The child had complete dietary tolerance and normal gastric biopsies at 6 months. He was transitioned to the local adult service and remains in remission from CD and EGID on 8 weekly vedolizumab and benralizumab therapies. A timeline of disease course and activity is summarized (Figure 2).

## 3 DISCUSSION

We report a unique case with terminal ileal CD who developed severe EGID and psoriasiform dermatitis in the context of anti–TNF therapy. The clinical course supports anti‐TNF–associated immune deviation with Th2‐skewed eosinophilic inflammation in a child with underlying atopic predisposition, rather than de novo primary EGID.^4^ Importantly, eosinophilic disease persisted despite anti‐TNF withdrawal, consistent with delayed immune recalibration and sustained downstream cytokine dysregulation described following TNF inhibition.

An in‐class switch from infliximab to ADA improved psoriatic skin disease, supporting a drug‐related mechanism, but had minimal impact on EGID symptoms or peripheral eosinophilia. Subsequent out‐of‐class transition to vedolizumab maintained mucosal remission of CD but did not control eosinophilic inflammation, highlighting the dissociation between Crohn's activity and EGID in this case. Despite cessation of anti‐TNF therapy for several months, tissue eosinophilia persisted, reinforcing the likelihood of ongoing immune deviation rather than active TNF‐mediated pathology.

Peripheral and tissue eosinophilia associated with anti‐TNF therapy has been described in small series and case reports,^6^, ^7^ with proposed mechanisms involving Th2 pathway activation and IL‐5–mediated eosinophil survival. IL‐5 targeted therapies reliably reduce tissue eosinophilia; however, clinical response varies across EGID subtypes,^8^, ^9^ underscoring the importance of individualized therapeutic selection. Although dupilumab has emerging evidence^10^ for efficacy in non‐eosinophilic esophagitis EGID, its mechanism relies on upstream cytokine modulation rather than direct eosinophil depletion, which, in our case, was considered less optimal in the setting of severe systemic and tissue eosinophilia. In this child, benralizumab led to normalization of eosinophil counts, marked symptomatic improvement, and histologic remission, and was well tolerated. Planned benralizumab withdrawal will require careful monitoring, as the durability of immune re‐equilibration following anti‐TNF‐associated deviation remains uncertain.

## 4 CONCLUSION

This rare pediatric application of combined vedolizumab and benralizumab illustrates the need for heightened awareness of paradoxical immune phenomena during biologic therapy, as optimal management strategies for refractory eosinophilic complications remain incompletely defined.

## CONFLICT OF INTEREST STATEMENT

The authors declare no conflicts of interest.

## ETHICS STATEMENT

Informed written consent was requested and provided by the child's family.
